# Direct measurement of surface photovoltage by AC bias Kelvin probe force microscopy

**DOI:** 10.3762/bjnano.13.63

**Published:** 2022-07-25

**Authors:** Masato Miyazaki, Yasuhiro Sugawara, Yan Jun Li

**Affiliations:** 1 Department of Applied Physics, Graduate School of Engineering, Osaka University, 2-1 Yamadaoka, Suita, Osaka 565-0871, Japan

**Keywords:** atomic force microscopy, Kelvin probe force microscopy, photocatalyst, surface photovoltage, titanium dioxide

## Abstract

Surface photovoltage (SPV) measurements are a crucial way of investigating optoelectronic and photocatalytic semiconductors. The local SPV is generally measured consecutively by Kelvin probe force microscopy (KPFM) in darkness and under illumination, in which thermal drift degrades spatial and energy resolutions. In this study, we propose the method of AC bias Kelvin probe force microscopy (AC-KPFM), which controls the AC bias to nullify the modulated signal. We succeeded in directly measuring the local SPV by AC-KPFM with higher resolution, thanks to the exclusion of the thermal drift. We found that AC-KPFM can achieve a SPV response faster by about one to eight orders of magnitude than classical KPFM. Moreover, AC-KPFM is applicable in both amplitude modulation and frequency modulation mode. Thus, it contributes to advancing SPV measurements in various environments, such as vacuum, air, and liquids. This method can be utilized for direct measurements of changes in surface potential induced by modulated external disturbances.

## Introduction

Surface photovoltage (SPV) is the change in surface potential caused by light illumination [1–2] and is measured to determine such features as band bending [3–4], the lifetimes of excited carriers [5–7], the minority carrier diffusion length [8–9], and the plasmonic effect [10–12]. The local SPV is usually measured by Kelvin probe force microscopy (KPFM) [13–21], which is based on atomic force microscopy (AFM) [22]. KPFM measures the contact potential difference (CPD), which corresponds to the difference in work function between the tip and the sample, consecutively in darkness and under illumination, to determine the SPV values: SPV = CPD_light_ − CPD_dark_. In this method, the thermal drift between darkness and illumination degrades the spatial and energy resolutions, reducing the accuracy of SPV measurements on the nanometer scale [23].

To overcome the above problem, direct SPV measurements by means of laser power modulation and a lock-in technique have been proposed. Streicher et al. used two tandem lock-in amplifiers, tandem SPV-KPFM [24], which can measure only slow SPV responses on the subsecond time scale because it uses closed-loop DC bias feedback on the millisecond-to-second time scale. Sugawara et al. used two parallel lock-in amplifiers, parallel SPV-KPFM [25], which also detects relatively slow SPV responses because it is based on the frequency modulation (FM) method in which bandwidth is limited to below a few kilohertz. Recently, a different type of methodology of directly measuring the SPV that is not based on a standard lock-in technique has been demonstrated and implemented to perform time-resolved measurements of SPV [23,26–27].

In this paper, we propose a novel method of directly measuring the local SPV, namely AC bias Kelvin probe force microscopy (AC-KPFM), which is based on a lock-in technique (widely used in standard KPFM setups) and controls the AC bias to nullify a modulated signal, referring to the AC bias null method presented by Kohl and co-workers [28]. AC-KPFM avoids the problem of thermal drift and achieves a higher resolution. We provide the theory for both the amplitude modulation (AM) mode [29] and the frequency modulation (FM) mode [30] and demonstrate experiments using AC-KPFM in the FM mode.

## Theory of AC-KPFM for SPV Measurements

### General concept

KPFM measures the CPD by compensating the electrostatic forces between the tip and the sample. When an AC bias *V*_AC_·cos(ω_m_*t*) with modulation frequency ω_m_ between the tip and the sample is applied, the electrostatic force *F*_ele_ in darkness is described as


**Figure 1:**
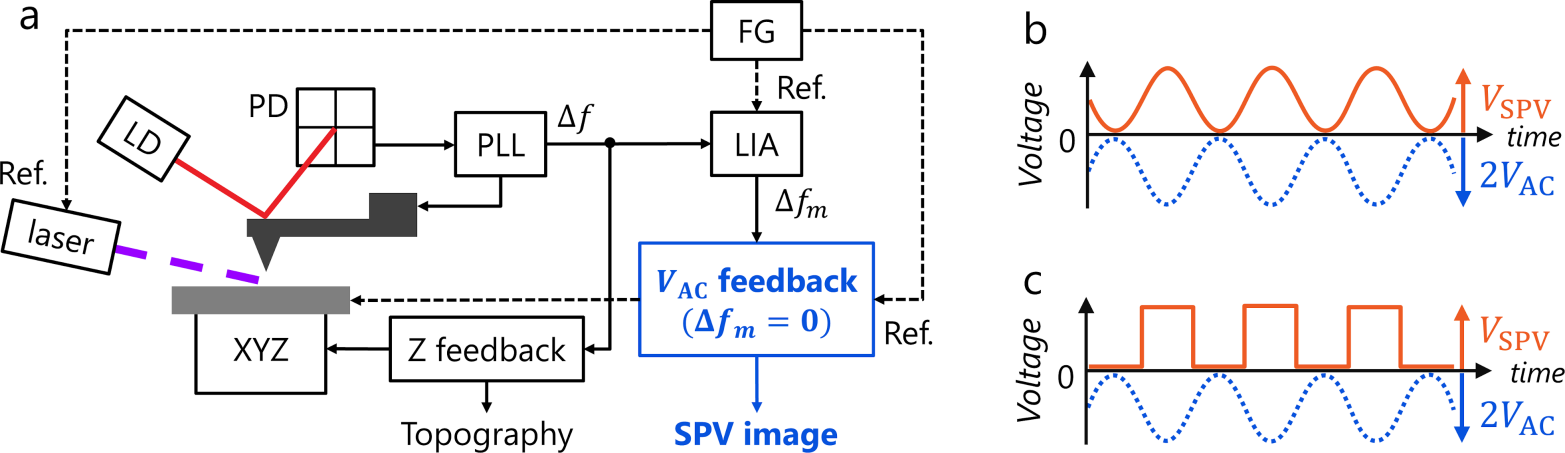
Schematics of AC-KPFM for direct SPV measurements. (a) Block diagram of AC-KPFM in FM mode. FG is a function generator. (b, c) Scheme of the AC bias nullifying method by laser power modulation with (b) sinusoidal and (c) square waveforms, which are synchronized to *V*_AC_.

[1] $$F_{\text{ele}}=\frac{1}{2}\frac{\partial C}{\partial z}{\left(V_{\text{DC}}+V_{\text{AC}}\cos\omega_{\text{m}}t-V_{\text{CPD}}\right)}^{2},$$

where ∂*C*/∂*z* is the capacitance gradient of the tip–sample system and *V*_CPD_ is the CPD in darkness. Applying a modulated laser power with a sinusoidal waveform of frequency ω_m_, which is synchronized with the AC bias (Figure 1b), induces the SPV with the peak-to-peak amplitude *V*_SPV_:


[2] $$f_{\text{SPV}}=\frac{1}{2}\left(1+\cos\omega_{\text{m}}t\right)V_{\text{SPV}}.$$

Therefore, the electrostatic force 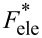 under modulated laser irradiation is described as


[3] $$\begin{array}{c} F_{\text{ele}}^{*}=\frac{1}{2}\frac{\partial C}{\partial z}(V_{\text{DC}}+V_{\text{AC}}\cos\omega_{\text{m}}t\\ {-\left\{V_{\text{CPD}}+\frac{1}{2}\left(1+\cos\omega_{\text{m}}t\right)V_{\text{SPV}}\right\})}^{2}. \end{array}$$

This equation can be divided into three parts:


[4] $$\begin{array}{c} F_{\text{DC}}^{*}=\frac{1}{2}\frac{\partial C}{\partial z}{\left(V_{\text{DC}}-V_{\text{CPD}}-\frac{1}{2}V_{\text{SPV}}\right)}^{2}\\ +\frac{1}{4}\frac{\partial C}{\partial z}{\left(V_{\text{AC}}-\frac{1}{2}V_{\text{SPV}}\right)}^{2} \end{array}$$

[5] $$\begin{array}{c} F_{\omega_{\text{m}}}^{*}=\frac{\partial C}{\partial z}\left(V_{\text{DC}}-V_{\text{CPD}}-\frac{1}{2}V_{\text{SPV}}\right)\\ \cdot \left(V_{\text{AC}}-\frac{1}{2}V_{\text{SPV}}\right)\cos\omega_{\text{m}}t \end{array}$$

[6] $$F_{2\omega_{\text{m}}}^{*}=\frac{1}{4}\frac{\partial C}{\partial z}{\left(V_{\text{AC}}-\frac{1}{2}V_{\text{SPV}}\right)}^{2}\cos2\omega_{\text{m}}t.$$

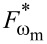
 (Equation 5) is measured to determine the SPV by controlling *V*_AC_ and nullifying the modulated force 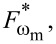 where the SPV is derived as


[7] $$V_{\text{SPV}}=2V_{\text{AC}}.$$

Thus, AC-KPFM controls the AC bias *V*_AC_ to directly measure the SPV, unlike classical KPFM, in which the DC bias *V*_DC_ is controlled to determine the CPD or SPV. It is noted that when the SPV is negative, *V*_AC_ yields a negative amplitude, where the phase of the AC bias is in phase opposition. It is also noted that it would be useful to measure the signal of the second harmonic component 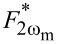 (Equation 6) since zero amplitude of 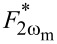 indicates that the SPV is correctly compensated by the *V*_AC_ control.

Next, when the laser power is modulated with a square waveform of frequency ω_m_ using, for example, a chopper synchronized to the AC bias (Figure 1c), the SPV with a peak-to-peak amplitude *V*_SPV_ is expressed as a Fourier series,


[8] $$f_{\text{SPV}}=\frac{1}{2}V_{\text{SPV}}+\frac{2V_{\text{SPV}}}{\pi }\sum_{n=1}^{\infty }\frac{1}{2n-1}\cos\left(2n-1\right)\omega_{\text{m}}t.$$

Therefore, the electrostatic force 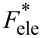 under square-waveform illumination is described as


[9] $$\begin{array}{c} F_{\text{ele}}^{*}=\frac{1}{2}\frac{\partial C}{\partial z}(V_{\text{DC}}+V_{\text{AC}}\cos\omega_{\text{m}}t-\{V_{\text{CPD}}+\frac{1}{2}V_{\text{SPV}}\\ +{\frac{2V_{\text{SPV}}}{\pi }\sum_{n=1}^{\infty }\frac{1}{2n-1}\cos\left(2n-1\right)\omega_{\text{m}}t\})}^{2}. \end{array}$$

The modulated force with frequency ω_m_ is described as


[10] $$\begin{array}{r} F_{\omega_{\text{m}}}^{*}=\frac{\partial C}{\partial z}\left(V_{\text{DC}}-V_{\text{CPD}}-\frac{1}{2}V_{\text{SPV}}\right)\\ \cdot \left(V_{\text{AC}}-\frac{2}{\pi }V_{\text{SPV}}\right)\cos\omega_{\text{m}}t. \end{array}$$

In the same manner as before, the SPV is determined by controlling *V*_AC_ and nullifying the modulated force 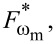



[11] $$V_{\text{SPV}}=\frac{\pi }{2}V_{\text{AC}}.$$

Thus, AC-KPFM can directly and quantitatively measure the SPV by laser power modulation with either sinusoidal or square waveforms.

### AC-KPFM in AM mode

In the AM mode, AC-KPFM measures the oscillation amplitude with frequency ω_m_, which is driven by the modulated electrostatic force 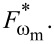 This signal is measured with a lock-in amplifier and compensated by *V*_AC_ control, yielding the SPV value. To improve the sensitivity, ω_m_ is usually tuned to the second (first) resonance frequency of the cantilever, while the first (second) resonance frequency is assigned to the AFM measurement [29]. Since these resonance frequencies are commonly in the kilohertz to megahertz range, the time scale of the measured SPV is from microseconds to milliseconds, which is much faster than that measured by classical KPFM, which measures the slow SPV response of the order of seconds to hours because of the long image acquisition time [31] and the need for consecutive experiments in darkness and under illumination. Here, ω_m_ should be set slower than the intrinsic SPV response, which we aim to observe, otherwise the SPV response cannot follow the modulated laser and yields zero amplitude. The spatial and energy resolutions and the image acquisition time of AC-KPFM in the AM mode are comparable to those of the classical KPFM in the AM mode, because both methods detect the electrostatic force, 
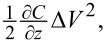
 and the response time of the bias feedback τ limits the image acquisition time. To reach sufficient sensitivity, the 
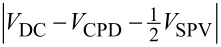
 value should typically be larger than 100 mV.

### AC-KPFM in FM mode

In the FM mode, AC-KPFM measures the modulated frequency shift 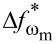 with frequency ω_m_, which is driven by the modulated electrostatic force 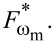 For a small oscillation amplitude, 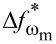 under a modulated laser can be approximately expressed as


[12] $$\begin{array}{c} \Delta f_{\omega_{\text{m}}}^{*}\propto \frac{\partial F_{\omega_{\text{m}}}^{*}}{\partial z}\\ =\frac{\partial^{2}C}{\partial z^{2}}\left(V_{\text{DC}}-V_{\text{CPD}}-\frac{1}{2}V_{\text{SPV}}\right)\\ \;\cdot \left(V_{\text{AC}}-\beta \cdot V_{\text{SPV}}\right)\cos\omega_{\text{m}}t, \end{array}$$

where


[13] $$\beta =\{\begin{array}{cc} \frac{1}{2}, & \text{sin wave laser}\\ \frac{2}{\pi }, & \text{square wave laser.} \end{array}$$

This signal is measured by a lock-in amplifier and compensated by controlling *V*_AC_, yielding the SPV value:


[14] $$\text{SPV}=\{\begin{array}{cc} 2V_{\text{AC}}, & \text{sin wave laser}\\ \frac{\pi }{2}V_{\text{AC}}, & \text{square wave laser.} \end{array}$$

Since ω_m_ is usually set in a range from a few tens of hertz to several kilohertz, the time scale of the measured SPV is of the order of milliseconds, which is faster than that measured by classical KPFM. The spatial and energy resolutions and the image acquisition time of AC-KPFM in the FM mode are comparable to those of the classical KPFM in the FM mode, because both methods detect the electrostatic force gradient 
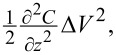
 and the response time of the bias feedback τ limits their image acquisition time [31]. To reach sufficient sensitivity, the 
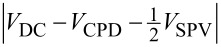
 value should typically be larger than 1 V.

## Experimental

The experiments were performed by customized ultrahigh-vacuum (UHV) noncontact atomic force microscopy (NC-AFM, UNISOKU) at a temperature *T* of 78 K with a base pressure below 5 × 10^−11^ Torr. The NC-AFM was operated in the FM mode [32] with a constant oscillation amplitude *A* of 500 pm. The cantilever deflection was measured by an optical beam deflection (OBD) method [33].

AC-KPFM was carried out in the FM mode, in which the topography and SPV were measured simultaneously. An AC bias *V*_AC_ with frequency ω_m_ and a DC bias *V*_DC_ were applied to the sample. The laser power was modulated to a square waveform by a chopper at frequency ω_m_, synchronized to the AC bias. *V*_AC_ is controlled to nullify the modulated frequency shift (Equation 12), yielding the SPV value. We used a digital lock-in amplifier (HF2LI with PID option, Zurich Instruments) to generate and control the AC bias. The typical sensitivity of our measurements was estimated to be δV = 1 mV (see Appendix). We simultaneously measured the tunneling current through the *I*/*V* converter as scanning tunneling microscopy (STM) [34] to consider the influence of the photocurrent [35–37].

The ultraviolet (UV) light source was a He–Cd laser (Kimmon Koha) with a wavelength of 325 nm and a laser power of 2 mW. A lens was equipped on a *xyz*-scanner in the UHV chamber to focus the laser onto the sample with a beam diameter of 500 µm. A band-pass filter was arranged in front of a photodetector of the OBD system to suppress the influence of the UV light on the deflection sensor.

We used a commercial Ir-coated Si cantilever (NANOSENSORS, SD-T7L100) with a resonant frequency *f*_0_ of 913 kHz, a spring constant *k* of 650 N/m, and a quality factor *Q* of 7748. The tip was cleaned by Ar^+^ sputtering (0.8 keV, 5 × 10^−7^ Torr, 5 min) to remove the contaminants and the native oxide layer. We used a rutile TiO_2_(110) sample to demonstrate the AC-KPFM. TiO_2_ is one of the promising photocatalytic materials [38–40] and has been widely studied using AFM and KPFM [41–44]. Rutile TiO_2_ has a bandgap of 3.0 eV [45] and shows the SPV under UV illumination [46–48]. A clean rutile TiO_2_(110) surface (Crystal Base) was prepared by several cycles of Ar^+^ sputtering (1 keV, 1 × 10^−6^ Torr, 15 min) and annealing (993 K, less than 2 × 10^−10^ Torr, 30 min).

## Results and Discussion

**Figure 2:**
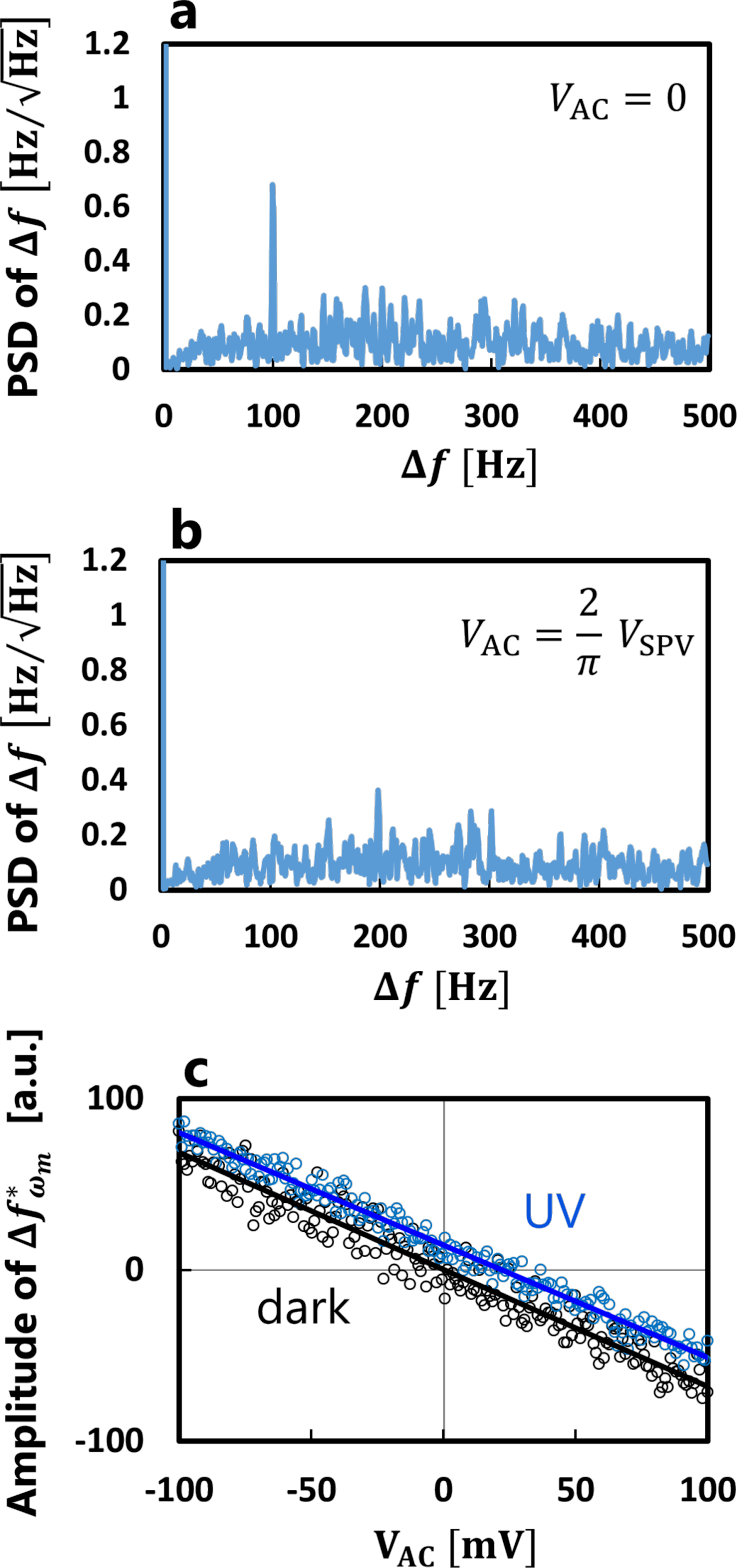
PSD of Δ*f* under modulated UV laser illumination (*f*_m_ = 100 Hz) with (a) *V*_AC_ = 0 and (b) *V*_AC_ = 2/π *V*_SPV_, where *V*_AC_ nullifies the SPV. (c) Dependence of the amplitude of 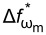 (the peak at 100 Hz in (a)) on *V*_AC_ in darkness (black) and under UV illumination (blue) measured using a lock-in amplifier. Circles and solid lines are experimental data and fit results, respectively.

First, we detected the SPV signal with AC-KPFM in the FM mode. Figure 2a shows the spectrum of the frequency shift Δ*f* under modulated UV laser illumination with frequency *f*_m_ = 100 Hz. The peak appeared at 100 Hz only when the tip approached the sample. Here, *V*_DC_ was set to −300 mV to ensure sufficient sensitivity (CPD ≈ 600 mV). Then we applied *V*_AC_, which was synchronized with the modulated laser, and measured the amplitude of that peak at 100 Hz depending on *V*_AC_ (Figure 2c). The amplitude of the peak showed linear behavior as a function of *V*_AC_ under UV illumination, which is consistent with Equation 12. While the *x*-intercept was 0 V in darkness, the *x*-intercept was 22.1 mV under UV illumination, at which *V*_AC_ nullified the modulated frequency shift and the SPV value |*V*_SPV_| of 34.7 mV was determined from Equation 14. Therefore, when *V*_AC_ was set to 22.1 mV, the peak at 100 Hz disappeared in the spectrum of Δ*f* (Figure 2b). Here, we cannot determine the polarity of the SPV because the phase for the lock-in amplifier was adjusted to maximize the absolute value of the demodulated output. We note that the response time of SPV on the rutile TiO_2_(110) surface is intrinsically sufficiently fast (of the order of nanoseconds) [46,49] compared with the modulation frequency of 100 Hz. Thus, we confirmed that the SPV signal of AC-KPFM agreed with the present theory described above.

**Figure 3:**
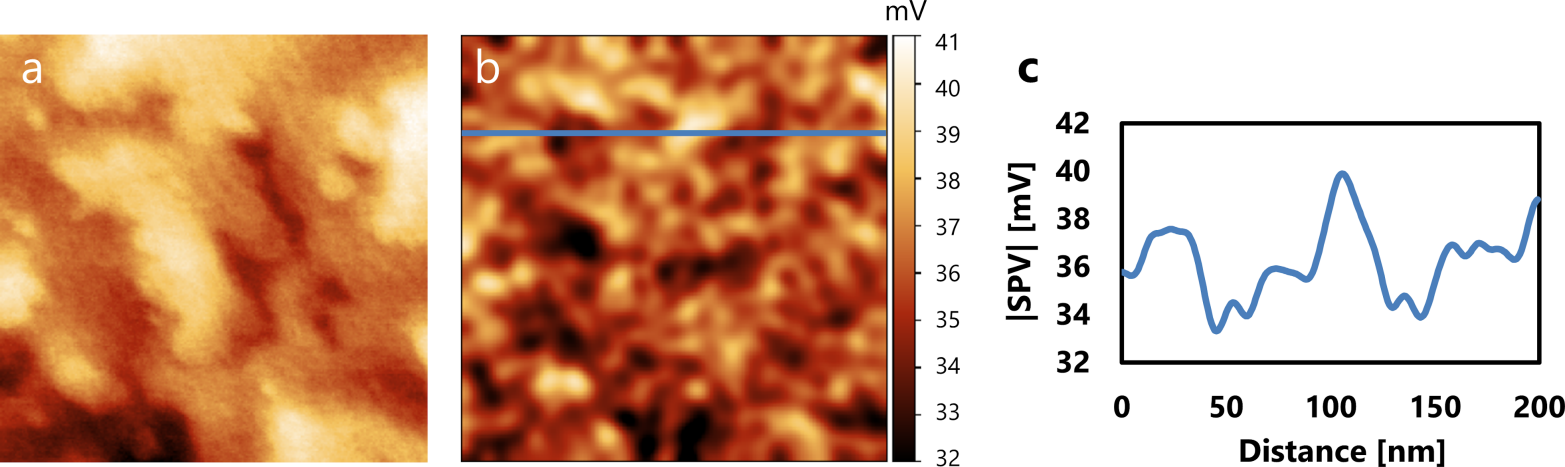
AC-KPFM imaging for SPV measurement. (a) Topographic and (b) SPV images of TiO_2_(110) surface. (c) SPV profile along the blue line in (b). The acquisition parameters are *f*_m_ = 100 Hz, Δ*f* = −80 Hz, *V*_DC_ = −0.3 V, and an imaging size of 200 × 200 nm^2^.

Next, we performed AC-KPFM imaging to directly obtain the SPV distribution. Figure 3a shows the AFM image of the rutile TiO_2_(110) surface. Terrace and step structures were observed, and the surface was flat within a single step height of 325 pm [50]. Figure 3b shows the SPV image obtained simultaneously with the AFM image. No tunneling current was detected in the AC-KPFM measurement. The SPV profile is shown in Figure 3c. The AC-KPFM successfully resolved the inhomogeneous SPV distribution with fluctuations on scales of 10–50 nm and a few millivolts, whereas classical KPFM observed a homogeneous SPV distribution over the TiO_2_ surface with sub-micrometer resolution [12,51] because of the influence of thermal drift between darkness and illumination. In the case of semiconductors, an electric field is screened on the scale of the Debye length *L*_D_ [3],


[15] $$L_{\text{D}}=\sqrt{\frac{k_{\text{B}}T\varepsilon_{0}\varepsilon_{\text{r}}}{e^{2}n}},$$

where *k*_B_ is the Boltzmann constant, *T* is the temperature, ε_0_ is the vacuum permittivity, ε_r_ is the relative permittivity of the semiconductor, *e* is the elementary charge, and *n* is the carrier density. The carrier density of TiO_2_ can be estimated from the crystal color and was 10^16^–10^18^ cm^−3^ for the slightly reduced TiO_2_ (light-blue color) [52–54]. At a temperature of 78 K, *L*_D_ is calculated to be 8–80 nm, while a ε_r_ value of rutile TiO_2_ of 170 is used [55–56]. The calculated *L*_D_ is consistent with the spatial extent of the local SPV observed by AC-KPFM. We could not find any correlation between the topographic and SPV images. The origin of the inhomogeneous SPV would involve the local distribution of the defect concentration, trapping sites, and an intrinsic electric field [57–59]; however, this is beyond the scope of this paper.

We note that the time scale of SPV measured with AC-KPFM is determined by the modulation frequency of the laser power and is faster (microseconds to milliseconds) than that in the case of classical KPFM (seconds to hours) because of the need for consecutive measurements in darkness and under illumination. Thus, AC-KPFM and classical KPFM measure the SPV derived from different origins, such as charge recombination (nanoseconds to milliseconds) [60], ion transport (milliseconds to seconds) [61], and surface chemical reactions (hours) [62–64]. Particularly for photocatalytic semiconductors, AC-KPFM would be an indispensable tool for detecting the fast SPV distribution related to charge redistribution (microseconds to milliseconds) because SPV measured with classical KFPM is attributed to both charge redistribution and surface modifications by photocatalytic reactions. Intriguingly, this fact implies that performing the AC-KPFM with changing the modulation frequency of the excitation laser between low and high frequencies is useful to determine the time constant of the SPV response and the origin of the SPV. Indeed, there remains an issue that the modulation frequency has the constraint of a transfer function of cantilever dynamics and the bandwidth of the PLL. We believe that this issue will be solved by future work such as using a heterodyne detection scheme [65–67]. It is noted that AC-KPFM observes not an instantaneous photo-response of the system but a photo-response in equilibrium states, which is synchronized to the modulation of the excitation laser. We are convinced that this SPV response also provides crucial and attractive information.

## Conclusion

We have proposed the method of AC bias KPFM (AC-KPFM), which nullifies the modulated SPV by controlling the AC bias, to directly measure the SPV distribution. It improves the spatial and energy resolutions thanks to the exclusion of the thermal drift between darkness and illumination, compared with the classical KPFM method. Moreover, we found that AC-KPFM can detect faster SPV responses (microseconds to milliseconds) depending on the modulation frequency of the laser power. AC-KPFM is applicable to both AM and FM modes, so it contributes to advancing SPV measurements in various environments such as vacuum, air, and liquids. Note that it would be useful to operate AC-KPFM with a heterodyne detection scheme [65–67] in order to reduce a photothermal effect on the cantilever dynamics [68–69] and measure the fast SPV phenomena. The AC-KPFM method is utilized not only for SPV measurements, but also for direct measurements of changes in surface potential induced by modulated external disturbances such as electric fields, magnetic fields [70–71], and stress fields [72–73].

## Appendix

### Sensitivity of AC-KPFM in the FM mode

The sensitivity of AC-KPFM in the FM mode is comparable to the sensitivity of FM-KPFM. The frequency noise density *n*_FM_ is described as [74]


[16] $$\begin{array}{c} n_{\text{FM}}={\left(n_{\text{therm}}^{2}+n_{\text{detect}}^{2}+n_{\text{osc}}^{2}\right)}^{\frac{1}{2}}\\ ={\left(\frac{k_{\text{B}}Tf_{0}}{\pi kQA^{2}}+\frac{2n_{\text{ds}}^{2}}{A^{2}}f_{\text{m}}^{2}+\frac{n_{\text{ds}}^{2}}{2Q^{2}A^{2}}f_{0}^{2}\right)}^{\frac{1}{2}}, \end{array}$$

where *n*_therm_ is the thermal noise density, *n*_detect_ is the detector noise density, *n*_osc_ is the oscillator noise density, and *n*_ds_ is the deflection sensor noise density. The frequency noise δ*f* measured with a bandwidth of *B* at a center of *f*_m_ is described as


[17] $$\begin{array}{c} \delta f={\left(\begin{array}{l} \int_{f_{\text{m}}-\frac{B}{2}}^{f_{\text{m}}+\frac{B}{2}}\frac{k_{\text{B}}Tf_{0}}{\pi kQA^{2}}\text{d}f_{m}+\int_{f_{\text{m}}-\frac{B}{2}}^{f_{\text{m}}+\frac{B}{2}}\frac{2n_{\text{ds}}^{2}}{A^{2}}f_{\text{m}}^{2}\text{d}f_{m}\\ +\int_{f_{\text{m}}-\frac{B}{2}}^{f_{\text{m}}+\frac{B}{2}}\frac{n_{\text{ds}}^{2}}{2Q^{2}A^{2}}f_{0}^{2}\text{d}f_{m} \end{array}\right)}^{\frac{1}{2}}\\ ={\left\{\frac{k_{\text{B}}Tf_{0}}{\pi kQA^{2}}B+\frac{2n_{\text{ds}}^{2}}{3A^{2}}\left(3f_{\text{m}}^{2}B+\frac{B^{3}}{4}\right)+\frac{n_{\text{ds}}^{2}}{2Q^{2}A^{2}}f_{0}^{2}B\right\}}^{\frac{1}{2}}. \end{array}$$

When *f*_m_ ≫ *B*, δ*f* is described as


[18] $$\delta f\approx {\left(\frac{k_{\text{B}}Tf_{0}}{\pi kQA^{2}}B+\frac{2n_{\text{ds}}^{2}}{A^{2}}f_{\text{m}}^{2}B+\frac{n_{\text{ds}}^{2}}{2Q^{2}A^{2}}f_{0}^{2}B\right)}^{\frac{1}{2}}.$$

Here, we note that the frequency noise derived from *n*_detect_ is proportional to *f*_m_ and small *f*_m_ yields a low noise level:


[19] $$\delta f_{\text{detect}}=\frac{\sqrt{2}n_{\text{ds}}}{A}f_{\text{m}}\sqrt{B}.$$

For a small oscillation amplitude, the measured signal strength 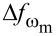 in AC-KPFM is approximately expressed as


[20] $$\begin{array}{c} \Delta f_{\omega_{\text{m}}}\approx \frac{f_{0}}{2k}\frac{\partial F}{\partial z}\\ =\frac{f_{0}}{2k}\frac{\partial^{2}C}{\partial z^{2}}\left(V_{\text{DC}}-V_{\text{CPD}}-\frac{1}{2}V_{\text{SPV}}\right)\\ \;\cdot \left(V_{\text{AC}}-\frac{2}{\pi }V_{\text{SPV}}\right)\cos\omega_{\text{m}}t. \end{array}$$

Therefore, the minimum detectable voltage δ*V* is described as


[21] $$\delta V\approx \frac{\delta f}{\frac{f_{0}}{2k}\frac{\partial^{2}C}{\partial z^{2}}\left(V_{\text{DC}}-V_{\text{CPD}}-\frac{1}{2}V_{\text{SPV}}\right)}.$$

**Figure 4:**
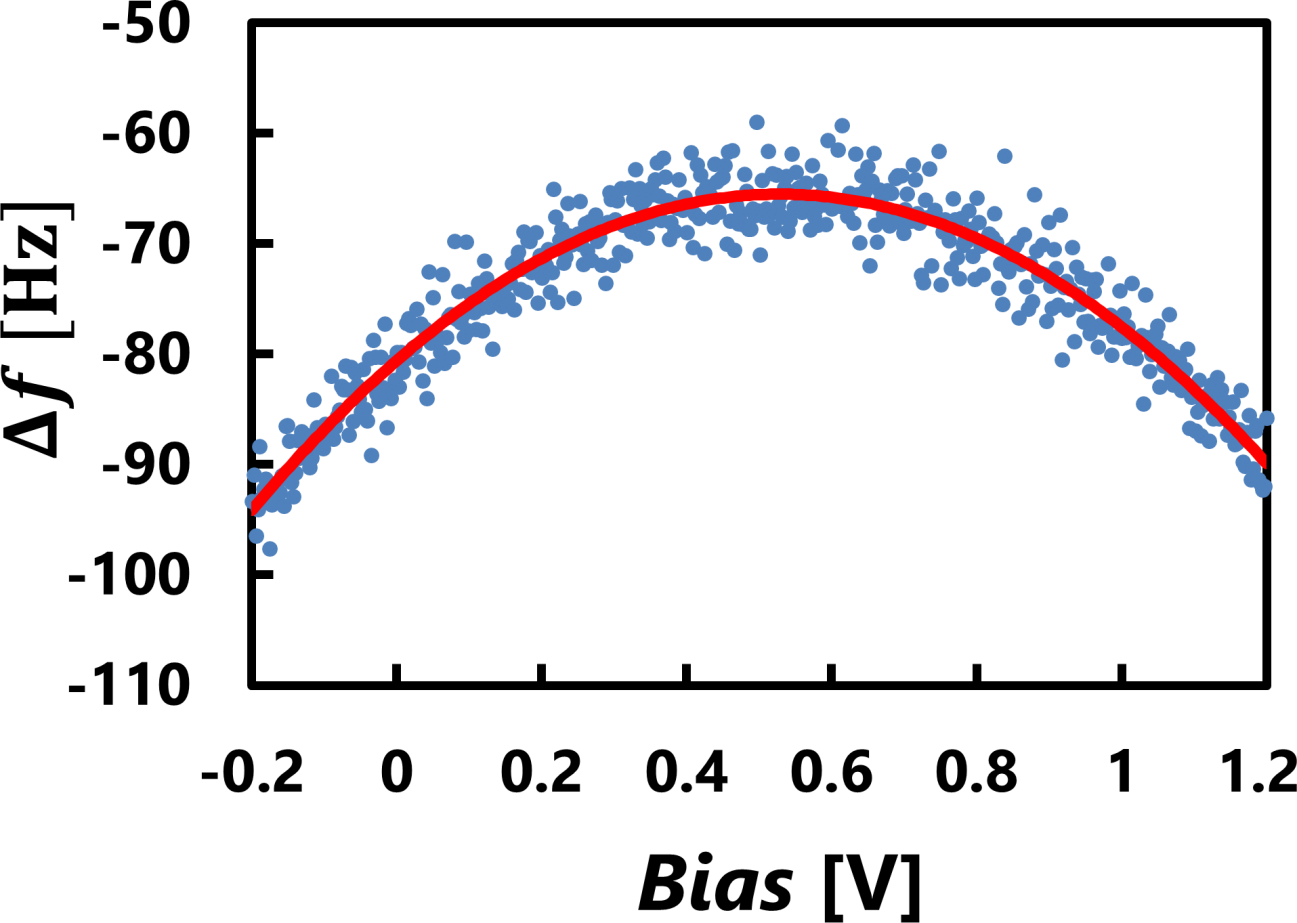
KPFS measurement on a TiO_2_(110) surface. KPFS data (blue dots) was fitted by Δ*f*(*V*) = *a*_1_*V*^2^ + *b*_1_ (red solid line), where 2*a*_1_ provides the value of 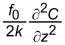.

The typical value of δ*f* and δ*V* in our measurements were estimated to be 0.1 Hz and 1 mV, respectively, with *T* = 80 K, *Q* = 10,000, *k* = 650 N/m, *A* = 500 pm, *f*_0_ = 910 kHz, *f*_m_ = 100 Hz, *B* = 10 Hz, *n*_ds_ = 100 fm/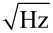, 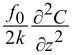 = 111.25 Hz/V^2^, and 
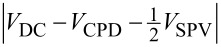
 = 1 V. The value of 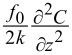 was calculated from Kelvin probe force spectroscopy (KPFS) [75–76], as shown in Figure 4.
